# Anti-inflammatory effects of antibacterials on human bronchial epithelial cells

**DOI:** 10.1186/1465-9921-10-89

**Published:** 2009-09-29

**Authors:** Gregor S Zimmermann, Claus Neurohr, Heidrun Villena-Hermoza, Rudolf Hatz, Juergen Behr

**Affiliations:** 1 Department of Internal Medicine I, Division of Pulmonary Diseases, Ludwig Maximilians University, Klinikum Grosshadern, Munich, Germany; 2 Division of Thoracic Surgery, Ludwig-Maximilians-University, Klinikum Grosshadern, Munich, Germany

## Abstract

**Background:**

Human Bronchial epithelial cells (hu-BEC) have been claimed to play a significant role in the pathogenesis of chronic inflammatory airway diseases like COPD. In this context IL-8 and GM-CSF have been shown to be key cytokines. Some antibiotics which are routinely used to treat lower respiratory tract infections have been shown to exert additional immunomodulatory or anti-inflammatory effects. We investigated whether these effects can also be detected in hu-BEC.

**Methods:**

Hu-BEC obtained from patients undergoing lung resections were transferred to air-liquid-interface (ALI) culture. These cultures were incubated with cefuroxime (CXM, 10-62.5 mg/l), azithromycin (AZM, 0.1-1.5 mg/l), levofloxacin (LVX, 1-8 mg/l) and moxifloxacin (MXF, 1-16 mg/l). The spontaneous and TNF-α (10 ng/ml) induced expression and release of IL-8 and GM-CSF were measured using PCR and ELISA in the absence or presence of these antibiotics.

**Results:**

The spontaneous IL-8 and GM-CSF release was significantly reduced with MXF (8 mg/l) by 37 ± 20% and 45 ± 31%, respectively (both p < 0.01). IL-8 release in TNF-α stimulated hu-BEC decreased by 16 ± 8% (p < 0.05) with AZM (1.5 mg/l). With MXF a concentration dependent decrease of IL-8 release was noted up to 39 ± 7% (p < 0.05). GM-CSF release from TNF-α stimulated hu-BEC was maximally decreased by 35 ± 24% (p < 0.01) with MXF (4 mg/l).

**Conclusion:**

Using ALI cultures of hu-BEC we observed differential effects of antibiotics on spontaneous and TNF-α induced cytokine release. Our data suggest that MXF and AZM, beyond bactericidal effects, may attenuate the inflammatory process mediated by hu-BEC.

## Background

Antimicrobial agents of different classes - e.g. betalactames, quinolones, and macrolides - are standard of care in the treatment of respiratory tract infections. In addition to their antimicrobial activity some of these antibiotics, especially macrolides and fluoroquinolones, have immunomodulatory effects [1-3]. These anti-inflammatory or immunomodulatory capabilities have been demonstrated in human cells, cell lines, and in animal experiments [1,4-7].

Due to intracellular accumulation of macrolides and quinolones in lung cells and in alveolar macrophages a targeted modulation of the inflammatory reaction could be of additional therapeutic benefit by attenuation of the inflammatory process in lower respiratory tract infection (LRTI) as well as in chronic non-infectious airway diseases like COPD [8-10].

Airway epithelial cells have been shown to be of crucial importance in the pathogenesis of inflammatory airway diseases [11]. In addition to antimicrobial activities, macrolides directly affect pulmonary host defence like the neutrophil activation and the immune cell function. These effects are mediated by an alteration of cytokine and chemokine release, as has been demonstrated in vitro and ex vivo [2,12]. Moreover, macrolides like azithromycin are already clinically used in chronic respiratory diseases like diffuse panbronchiolitis (DPB), cystic fibrosis despite they have no antimicrobial activity against Pseudomonas aeruginosa. A beneficial effect on bacterial virulence factors by inhibiting quorum-sensing, a mechanism of bacterial communication, is described for macrolides and quinolones as well [13-17].

Additionally, immunomodulatory effects of macrolides are used in bronchiolitis obliterans syndrom after bone marrow transplantation and lung transplantation which are diseases without infectious background [12,18,19]. There are many studies, which elucidated the immunomodulatory effects of macrolides in human cells [20,21]. However, the underlying intracellular mechanisms of immunomodulation by macrolides are not completely understood yet [20,21].

Similarly to macrolides, immunomodulatory effects have been shown for fluorquinolones in a variety of cells of the immune system and in lung epithelial cells. These effects were especially pronounced in fluorquinolones with a cyclopropyl-moiety at position N1 like ciprofloxacin and moxifloxacin [1]. Moreover, expression of pro-inflammatory cytokines in human monocytes is suppressed by moxifloxacin *in vitro *and *in vivo *in an animal model of inflammation [4,7]. Beside the modulation of cytokine release from cells of the immune system it has been shown, that quinolones reduce pro-inflammatory activities of respiratory epithelial cell lines, thus potentially influencing pulmonary host defence [5,6].

Therefore, we investigated the modulation of cytokine release from primary human bronchial epithelial cells in air-liquid interface culture by different antibiotics.

## Methods

### Preparation of air-liquid interface cultures of human bronchial epithelial cells (hu-BEC)

**Figure 1:**
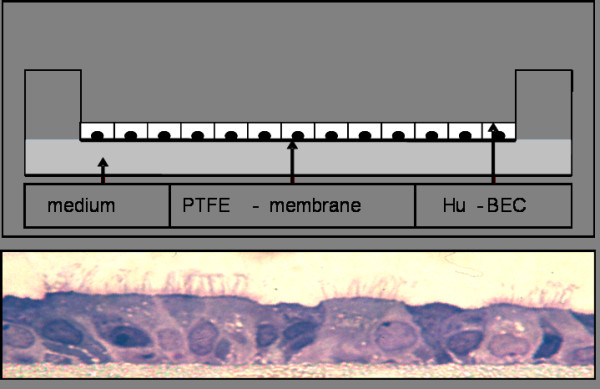
**air-liquid-interface-culture (schematic); HE-stain of an air-liquid-interface-culture with characteristic polarity**.

The human bronchial epithelial cells were harvested from patients undergoing lung surgery for cancer resection or transplantation [22,23]. Written informed consent was obtained from each patient according to the recommendations of the local ethic committee and there was an approval of our institutional review board. After preparation the resected bronchi were incubated for 24 h at 4°C in DMEM (Dulbeccos Modified Eagle Medium, Invitrogen, USA) and DTT (Dithio-Threitrol, Invitrogen, USA) containing penicillin G (Jenapharm, Germany), streptomycine (Rotexmedica, Germany), gernebcin (Infectopharm, Germany), imipenem (MSD, Germany) and amphotericin b (Bristol-Myer-Sqibb, Germany). Thereafter the bronchi were treated with protease Type XIV (Sigma, Germany) for 24 h at 4°C and rinsed several times with DMEM to wash out the epithelial cells. Then the cells were grown to 80% confluence with airway epithelial cell growth medium (Promocell, Germany) and after treatment with trypsin (0.05%, Invitrogen, USA) the cells were transferred on a collagenised PTFE membrane (polytetrafluorethylen, Millipore, USA) of 6-well plates (Corning Costar, USA) at a concentration 2 × 10^6 ^cells/ml and grown with DMEM containing HAM-12 (Invitrogen, USA), Ultroser G (Pall Life Sciences, France) and antibiotics (penicillin 100 U/ml and streptomycin 100 μg/ml, Invitrogen) at 37°C in 5% carbon dioxide/air. The supernatant was removed after 2 days and the cells were air-lifted. After another 14.0 ± 2.6 days these air-liquid-interface cultured cells expressed their characteristic bronchial polarity (see Fig. 1). Cultures were considered confluent and differentiated if the Rt was stable and > 500 Ω/cm^2 ^measured by Ohmmeter (EVOM, World Precision Instruments, USA).

### Incubation experiments

To characterize spontaneous cytokine-expression and release of hu-BEC, we incubated air-liquid-interface (ALI) cultures with buffer or with cefuroxime (62.5 mg/l), azithromycin (1.5 mg/l), levofloxacin (8 mg/l), and moxifloxacin (8 mg/l) for 24 h. Thereafter, the basolateral medium of each well was collected and frozen at -20°C. The cells were lysed with Trizol (GIBCO, Germany) and the lysates were stored at -80°C.

To investigate cytokine-expression and release of hu-BEC under pro-inflammatory conditions the ALI cultures cells were pre-incubated for 24 hours with buffer or with various antibiotics at different concentrations (see Table 1) and stimulated with TNF-alpha (10 ng/ml) for another 24-h incubation. Thereafter, the basolateral medium of each well was collected and frozen at -20°C. The cells were lysed with Trizol reagent (GIBCO, Germany) and the lysates were frozen at -80°C.

**Table 1:** Concentrations of cefuroxime (CXM), azithromycin (AZM), levofloxacin (LVX) and moxifloxacin (MXF) used for incubation experiments

	Concentration	TNF-α(10 ng/ml)	IL-8	GM-CSF	IL-8 PCR	GM-GSF PCR
CXM	10 mg/l	+	n = 11	n = 11	-	-
	25 mg/l	+	n = 17	n = 17	-	-
	62.5 mg/l	+	n = 11	n = 11	n = 11	n = 10
	62.5 mg/l	-	n = 11	n = 11	n = 11	n = 10

AZM	0.5 mg/l	+	n = 12	n = 10	-	-
	1.0 mg/l	+	n = 12	n = 10	-	-
	1.5 mg/l	+	n = 12	n = 10	n = 10	n = 11
	1.5 mg/l	-	n = 11	n = 8	n = 11	n = 11

LVX	1 mg/l	+	n = 12	n = 11	-	-
	4 mg/l	+	n = 16	n = 18	-	-
	8 mg/l	+	n = 11	n = 11	n = 11	n = 11
	8 mg/l	-	n = 10	n = 11	n = 11	n = 11

MXF	1 mg/l	+	n = 24	n = 22	-	-
	4 mg/l	+	n = 25	n = 23	-	-
	8 mg/l	+	n = 23	n = 23	-	-
	16 mg/l	+	n = 14	n = 14	n = 14	n = 14
	8 mg/l	-	n = 11	n = 11	n = 11	n = 11

To determine, whether there is a concentration-dependent effect of these antibiotics, we used a range of concentrations (see Table 1), which are reached in humans *in vivo *covering therapeutic levels in human serum, in bronchoalveolar lavage fluid, or in bronchial tissue [8,9].

Moxifloxacin and azithromycin was a generous gift from Bayer Healthcare Germany and Pfizer Germany. Cefuroxime and levofloxacin were purchased form Sanofi-Aventis (France) and DeltaSelect (Germany), respectively.

### ELISA

IL-8 and GM-CSF were measured in basolateral medium using enzyme-linked immunosorbent assays (ELISA) (both R&D Systems, USA) as previously described [24].

### RNA Extraction

RNA was extracted with Trizol according to the methods recommended by the manufacturer and frozen at -80°C. For analysis frozen epithelial cell lysates were re-dissolved in water. Total RNA yield was calculated by measuring the absorbance at 260 and 280 nm (assuming that A_260 _of 1 = 40 μg RNA). RNA integrity was judged by determining the ratio of A_260_/A_280_. Only samples with an A_260_/A_280 _ratio from 1.6 to 2.0 were used for the subsequent measurements.

### First-strand complementary deoxyribonucleic acid synthesis by reverse transcription

The RNA was transferred in cDNA with the cDNA synthesis kit (Fermentas, Germany) following the instruction of the manufacturer. The first-strand cDNA was stored at -80°C.

### Semiquantitative polymerase chain reaction

A sample of 1 μl of cDNA was used for each 20 μl PCR reaction. Primer sets used for the amplification of cytokines and the housekeeping gene glyceraldehyde-3-phosphate dehydrogenase (GAPDH) were as follows:

GAPDH (MWG-Biotech, Germany): Forward: 5'-TGA AGG TCG GAG TCA ACG GAT TTG GT-3'; Reverse: 5'-CAT GTG GGC CAT GAG GTC CAC CAC-3', (size of PCR product: 900 base pair [bp]).

IL-8 (MWG-Biotech, Germany): Forward: 5'-ATT TCT GCA GCT CTG TGT GAA-3'; Reverse: 5'-TGA ATT CTC AGC CCT CTT CAA-3', (size of PCR product: 255 bp).

GM-CSF (MWG-Biotech, Germany): Forward: 5'-ACA CTG CTG CTG AGA TGA ATG AAA CAG TAG-3', Reverse: 5'-TGG ACT GGC TCC CAG CAG TCA AAA GGG ATG-3', (size of PCR product: 286 bp).

Each 50- μl reaction mixture consisted of 5 μl of 10× PCR buffer, 1.5 μl MgCl_2 _(~1.5 mM), 1 μl of 10 mM dNTP mix, 5 μl of specific primer for GAPDH, the mediators (synthesized by MWG-Biotech, Germany) (~10 μM), 0.25 μl of *Taq *DNA Polymerase (GIBCO, Germany) (~2 U), and 37.25 μl of H_2_O. The cycles (Peltier Thermal Cycler, MJ Research, USA) used were as follows: GAPDH: 94°C for 3 min/94°C for 45 sec/60°C for 30 sec/72°C for 90 sec for 25 cycles, followed by an extension step of 10 min at 72°C. The same cycle conditions were used for the mediators. The annealing temperature and PCR cycles for the mediators were as follows: IL-8 58°C for 35 cycles; GM-CSF 65°C for 40 cycles.

Products of amplification were transferred on a 2% agarose gel and after electrophorese viewed using a 300-nm ultraviolet transluminator (Cybertech, Germany). Samples from RT reactions that did not contain RT served as negative controls. For quantification, PCR bands were stained with ethidium bromide (Sigma, Germany) and signal intensity was measured with an ultraviolet densitometer (Cybertech, Germany). Densitometric values are expressed as the ratio of IL-8/GAPDH and GM-CSF/GAPDH.

### Statistical analysis

All statistical analyses were performed with SPSS 11.5 (Chicago, USA). The results are expressed as mean values ± SEM. We applied a non-parametric Wilcoxon-Test in our exploratory analysis. Conventionally, p < 0.05 was considered significant. The correlations of the data obtained by ELISA and PCR were calculated using the Pearson's test.

## Results

### Effects on spontaneous IL-8 release

**Figure 2:**
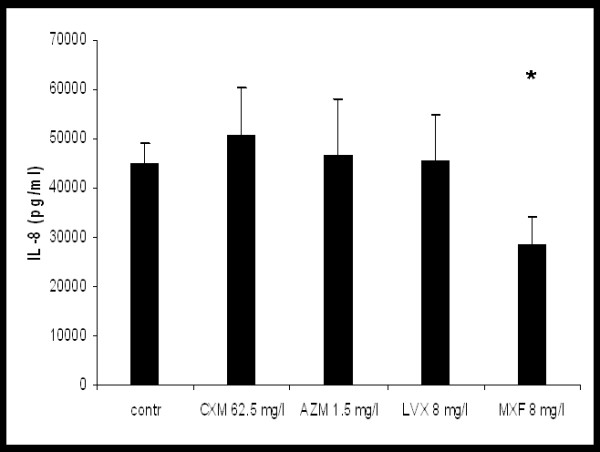
Effect of cefuroxime (CXM), azithomycin (AZM), levofloxacin (LVX) and moxifloxacin (MXF) on spontaneous IL-8 release from hu-BE,*p < 0.05 vs. control.

Spontaneous IL-8-release of hu-BEC in ALI cultures was 44.7 ± 4.3 ng/ml. No significant changes were observed with CXM (62.5 mg/l), AZM (1.5 mg/l), and LVX (8 mg/l). After 24 h incubation with MXF (8 mg/l) IL-8 release was reduced by 37 ± 20% (p < 0.008) (fig. 2).

### Effects on TNF-α stimulated IL-8 release

**Figure 3:**
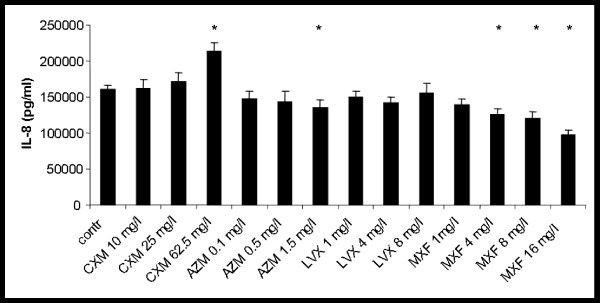
Effect of cefuroxime (CXM), azithomycin (AZM), levofloxacin (LVX) and moxifloxacin (MXF) on TNF-α-stimulated IL-8-release; * p < 0.05 vs. control.

Stimulation with TNF-α resulted in a 3.4-fold increase of IL-8 release to 160.2 ± 6.4 ng/ml (p < 0.001). Incubation with cefuroxime at a concentration of 62.5 mg/l led to a significant further increase of IL-8 release in stimulated hu-BEC by 33 ± 6% (p < 0.013). Under stimulated conditions azithromycin showed a significant reduction of IL-8 production up to 16 ± 8% at a concentration of 1.5 mg/l (p < 0.016). No significant changes were observed with levofloxacin at concentrations of 1, 4, and 8 mg/l. Incubation with moxifloxacin led to a concentration dependent reduction of IL-8 release to a maximum of 39 ± 7% (p < 0.001) at a concentration 16 mg/l (see Fig. 3).

### Effects on spontaneous GM-CSF release

**Figure 4:**
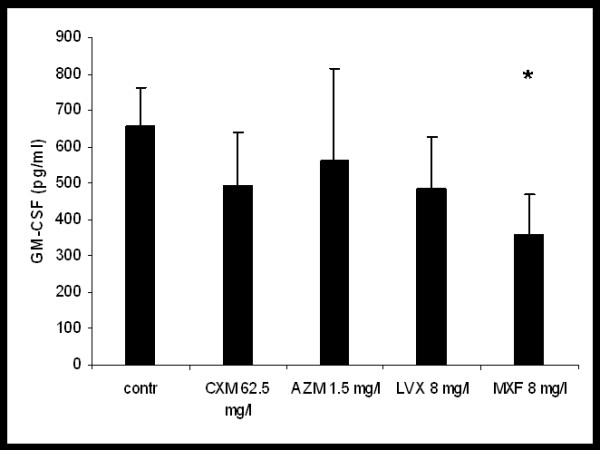
Effect of cefuroxime (CXM), azithomycin (AZM), levofloxacin (LVX) and moxifloxacin (MXF) on spontaneous GM-CSF release from hu-BE,*p < 0.05 vs. control.

Spontaneous GM-CSF-release of hu-BEC in ALI cultures was 654 ± 108 pg/ml. Incubation with CXM, AZM, or LVX did not show a significant effect on GM-CSF release with all concentrations tested. Only MXF reduced GM-CSF release by 45 ± 31% (p < 0.004) (see Fig. 4).

### Effects on TNF-α stimulated GM-CSF release

**Figure 5:**
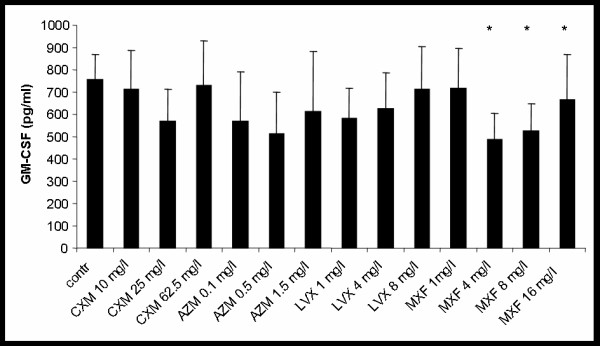
Effect of cefuroxime (CXM), azithomycin (AZM), levofloxacin (LVX) and moxifloxacin (MXF) on TNF-α-stimulated GM-CSF-release; * p < 0.05 vs. control.

Stimulation with TNF-α did not significantly alter GM-CSF release from hu-BEC in ALI cultures (maximum effect +17 ± 7%, n.s.). GM-CSF release of TNF-α stimulated hu-BEC in ALI cultures was also not significantly influenced by incubation with different concentrations of CXM, AZM, or LVX. Only MXF inhibited GM-CSF release in TNF-α-stimulated hu-BEC with an inverse concentration response characteristic (fig. 5). MXF concentration of 4 mg/l reduced GM-CSF release by 35 ± 24% (p < 0.009), MXF 8 mg/l reduced GM-CSF release by 30 ± 23% (p < 0.013), and MXF 16 mg/l reduced GM-CSF release by 22 ± 31% (p < 0.019) (fig. 5).

### PCR Analyses

Spontaneous IL-8 mRNA/GAPDH ratio was 1.2 ± 0.06 in the semi-quantitative PCR. The IL-8 mRNA/GAPDH ratio was reduced by 21 ± 8% after incubation with 8 mg/l MXF in unstimulated cells. Smaller effects were observed with CXM, AZM, or LVX (all n.s.).

In TNF-α stimulated hu-BEC IL-8 mRNA/GAPDH ratio increased to 1.54 ± 0.06 (p < 0,001). Incubation with CXM, AZM, LVX, or MXF led to maximal changes of IL-8/GAPDAH ratio of -15 ± 8%, +5 ± 12%, -8 ± 10%, and -11 ± 7%, respectively (all n.s.).

The spontaneous and TNF-α stimulated GM-CSF/GAPDH-ratio of hu-BEC in ALI cultures was 1.64 ± 0.08 and 1.81 ± 0.08, respectively. Incubation with CXM, AZM, LVX, or MXF did not significantly alter GM-CSF/GAPDH-ratio at all concentrations investigated (n.s.).

Correlation analysis revealed weak correlations between IL-8 protein as measured by ELISA and IL-8 m-RNA/GAPDH ratio (r = 0.373, p < 0.001) as well as between GM-CSF protein and GM-CSF mRNA/GAPDH ratio (r = 0.209; p < 0.004).

## Discussion

The data presented here demonstrate that some antibiotics are capable of modifying the inflammatory activation of human bronchial epithelial cells. The differential effects observed between different groups of antibiotics suggest that the member of the cephalosporine group cefuroxime do not show this effect, whereas azithromycin and moxifloxacin exert anti-inflammatory effects on hu-BEC, with moxifloxacin suppressing IL-8 and GM-CSF with and without TNF-α stimulation in our experimental setting, whereas AZM decreased IL-8 only after stimulation with TNF-α and had no significant effect on GM-CSF.

Immunomodulatory effects of antibiotics have been described so far *in vivo *with animal models, *in vitro *with models of immune cells, NHBE cells (normal human bronchial epithelial cells) and immortalised respiratory cell lines [1,4-7]. In those experiments it could be demonstrated that MXF leads to a reduction of IL-8, TNF-α, IL-1α, IL-1β, IL-4 and IFN-γ in monocytes, lymphocytes and neutrophils after stimulation with different agents. The direct effect on GM-CSF has not been investigated yet in cells of the immune system. However, in a mouse model of bone-marrow ablation with cyclophosphamide MXF leads to an increase of WBC and GM-CSF was augmented in the lungs of these mice [25]. In contrast, our findings suggest that MXF reduces spontaneous and TNF-α stimulated GM-CSF production and release of hu-BEC. This could be related to the different models and the different stimuli used.

For lung cells, only data of A549 cells, an immortalized type II alveolar epithelial cell line and IB3 cells, a cystic fibrosis cell line, are published [5,6]. In IB 3 cells MXF reduced the release of IL-8 and other cytokines [6]. In A549 cells MXF decreases NO production and NF-κB-activation [5]. Our study demonstrates anti-inflammatory effects of quinolones in a human *ex vivo *model of primary bronchial epithelial cells. The concentrations of the different antibiotics employed were comparable to concentrations reached by therapeutic medication in humans. Using primary hu-BEC in ALI cultures and therapeutically relevant concentrations of different antibiotics suggest that these findings may be also clinically relevant and may have implications for the treatment of human lung diseases.

In our study, we investigated the effect of different antibiotics on IL-8 and GM-CSF after application of TNF-α as an inflammatory stimulus. TNF-α is a proinflammatory cytokine with pro-fibrotic features which has a key role in lower respiratory tract infections as well as in chronic inflammatory lung disease like asthma, bronchiolitis obliterans, or COPD [11,26,27]. A blockade of TNF-α led to decrease of IL-8 after stimulation with LPS in lungs of patient with COPD [27].

Similarly, IL-8 and GM-CSF are key mediators not only in acute infectious inflammation but also in chronic inflammation as observed in COPD, bronchial asthma, and bronchiolitis obliterans [24,26,28]. IL-8 is rapidly induced by an inflammatory stimulus like TNF-α or LPS and is one of the most potent neutrophil chemoattractants in human tissue [27,29]. GM-CSF leads to an activation and increased survival of leukocytes and enhance oxidative burst in the lungs, thus maintaining and prolonging inflammatory reactions [28].

As we and other have shown, IL-8 and GM-CSF are secreted locally by the respiratory epithelium [24-26,28,30]. However, there is no specific treatment yet in humans to directly address and modify these cytokines to suppress the inflammatory cascade.

Our observations suggest that some antibiotics may have the capability to block or modulate this inflammation. In our experiments we employed concentrations of AZM, LVX and MXF which were comparable to therapeutic concentrations of these antibiotics and are reached in human lungs in vivo [8,9]. The serum level after therapeutic doses of MXF and LVX is 1-5 mg/l and after oral administration concentrations reached in the epithelial lining fluid (ELF) are 5 - 7 times higher than serum levels [9]. After oral administration with AZM serum level is 0.10 mg/l and the concentration in the ELF ranges from 0.94 mg/l to 1.2 mg/l after oral administration [8]. However, AZM accumulates intracellulary in alveolar macrophages with a concentration of 205.24 mg/l 24 hours after the last intake under steady state conditions [8,10]. The concentration of cefuroxime used in our experiments covers a range of serum and intrapulmonary concentrations after oral and continuous i.v. administration in humans [31-34]. Additionally we used a concentration of cefuroxime (62.5 mg/L) above these therapeutic intrapulmonary concentrations.

So far AZM and other macrolides are the only antibiotics used for therapeutic modulation of the local immune system in the lung. A beneficial effect of AZM has been demonstrated in the management of cystic fibrosis lung disease and diffuse panbrochiolitis (DPB) [2,3,18]. DPB is a disease observed predominantly in Asia, which without medical intervention leads to a rapid decline of lung function and death [3]. AZM is also used for treatment of bronchiolitis obliterans after organ transplantation, a chronic inflammatory and fibroproliferative disease leading to bronchiolar obstruction and obliteration of distal airspaces after lung transplantation but also after haematopoetic stem cell transplantation [35,36]. In our experiments only AZM at a concentration of 1.5 mg/l was associated with a significant reduction of IL-8 release. These findings differ from results in NHBE cells, a human bronchial epithelial cell line, where AZM at a concentration of 1.0 mg/l did not show an effect, whereas at a concentration of 10 mg/l an increase in IL-8-secretion was observed [37]. However, *in vivo *a concentration of 10 mg/l cannot be found under steady state conditions in ELF of the normal lung and was, therefore, not investigated in our experiments with hu-BEC. Hence, the immunomodulatory effects mediated by macrolides may not only depend on a direct effect on lung epithelial cells, but also on a direct effect on alveolar macrophages because of the intracellular accumulation in alveolar macrophages.

We also investigated effects on IL-8 mRNA and GM-CSF mRNA expression. In general the mRNA expressions of both, IL-8 and GM-CSF, were correlated with IL-8 and GM-CSF protein release, thus supporting the view that changes in protein release were related to changes in gene expression. However, the differences in mRNA expression between different experimental groups were not statistical significant. This could be due to the fact that changes of gene expression may be transient and are less well detected after 24-hours of incubation, when the cells were lysed and the mRNA isolated. In this respect further studies are needed to quantify the effect on mRNA-levels at earlier time points.

Although our data suggests that quinolones exert anti-inflammatory effects on hu-BEC, these effects are not uniform for all quinolones. In our experiments, moxifloxacin, a quinolone with a cyclopropyl-moiety at N1 (like ciprofloxacin) had a more pronounced effect on cytokine release when compared to levofloxacin, a quinolone lacking this cyclopropyl-moiety at N1 [1]. Despite the above-mentioned anti-inflammatory effects a careful use of quinolones is recommended due to risk of cross-resistance.

Several intracellular signal transduction pathways mechanisms are thought to be responsible for these anti-inflammatory effects [1,4-6]. Yet these mechanisms are not completely understood. Previous studies have shown that pre-treatment with MXF leads to an inhibition of the MAP-Kinases ERK 1/2 and JNK in monocytes [4,38]. MXF also inhibits the phosphorylation of these kinases in IB3 cells, C38 cells and A549 cells [5,6]. In contrast, the MAP-kinase p38 was not influenced by MXF [6]. Additionally, in monocytes and respiratory cell lines MXF inhibits NF-κB-activation due to reduced Iκ-B degradation [38]. This prevents NF-κB activation and translocation to the nucleus and thus inhibits the cytokine cascade.

## Conclusion

Our data confirm previous studies showing a significant inhibitory effect of quinolones with a cyclopropyl-moiety at N1 on cytokine release. Our study adds new aspects by using primary hu-BEC in ALI cultures and by employing therapeutically relevant concentrations of different antibiotics. When compared to MXF, AZM showed smaller effects on IL-8 release and did not affect GM-CSF release in concentration which can be reached in human ELF. In contrast, LVX showed no significant effects on cytokine release and CXM led to an increase in IL-8 release. Therefore, MXF appears to be more potent as an anti-inflammatory substance in bronchial epithelial cells. However, the clinical relevance of these findings has not been evaluated yet.

## Competing interests

GSZ has received a travel fee and a fund for speaking at symposium organized on behalf of Bayer Healthcare in 2007. The other authors have none to declare.

## Authors' contributions

GSZ and HVH have carried out the experimental work. GSZ carried out the data analysis and drafted the manuscript. GSZ, JB and RH initiated the study and designed the experiments. CN participated in the design of the study. RH provided the surgical specimens. All authors read and approved the final version of the manuscript.

## References

[B1] Dalhoff A Shalit I Immunomodulatory effects of quinolones Lancet Infect Dis 2003 3 6 359 371 10.1016/S1473-3099(03)00658-3 12781508

[B2] Rubin BK Henke MO Immunomodulatory activity and effectiveness of macrolides in chronic airway disease Chest 2004 125 2 Suppl 70S 78S 10.1378/chest.125.2_suppl.70S 14872003

[B3] Schultz MJ Macrolide activities beyond their antimicrobial effects: macrolides in diffuse panbronchiolitis and cystic fibrosis J Antimicrob Chemother 2004 54 1 21 28 10.1093/jac/dkh309 15190022

[B4] Shalit I Halperin D Haite D Levitov A Romano J Osherov N Fabian I Anti-inflammatory effects of moxifloxacin on IL-8, IL-1beta and TNF-alpha secretion and NFkappaB and MAP-kinase activation in human monocytes stimulated with Aspergillus fumigatus J Antimicrob Chemother 2006 57 2 230 235 10.1093/jac/dki441 16352735

[B5] Werber S Shalit I Fabian I Steuer G Weiss T Blau H Moxifloxacin inhibits cytokine-induced MAP kinase and NF-kappaB activation as well as nitric oxide synthesis in a human respiratory epithelial cell line J Antimicrob Chemother 2005 55 3 293 300 10.1093/jac/dkh525 15659543

[B6] Blau H Klein K Shalit I Halperin D Fabian I Moxifloxacin but not ciprofloxacin or azithromycin selectively inhibits IL-8, IL-6, ERK1/2, JNK, and NF-kappaB activation in a cystic fibrosis epithelial cell line Am J Physiol Lung Cell Mol Physiol 2007 292 1 L343 352 10.1152/ajplung.00030.2006 17012372

[B7] Shalit I Horev-Azaria L Fabian I Blau H Kariv N Shechtman I Alteraz H Kletter Y Immunomodulatory and protective effects of moxifloxacin against Candida albicans-induced bronchopneumonia in mice injected with cyclophosphamide Antimicrob Agents Chemother 2002 46 8 2442 2449 10.1128/AAC.46.8.2442-2449.2002 12121916 PMC127325

[B8] Capitano B Mattoes HM Shore E O'Brien A Braman S Sutherland C Nicolau DP Steady-state intrapulmonary concentrations of moxifloxacin, levofloxacin, and azithromycin in older adults Chest 2004 125 3 965 973 10.1378/chest.125.3.965 15006955

[B9] Soman A Honeybourne D Andrews J Jevons G Wise R Concentrations of moxifloxacin in serum and pulmonary compartments following a single 400 mg oral dose in patients undergoing fibre-optic bronchoscopy J Antimicrob Chemother 1999 44 6 835 838 10.1093/jac/44.6.835 10590288

[B10] Lucchi M Damle B Fang A de Caprariis PJ Mussi A Sanchez SP Pasqualetti G Del Tacca M Pharmacokinetics of azithromycin in serum, bronchial washings, alveolar macrophages and lung tissue following a single oral dose of extended or immediate release formulations of azithromycin J Antimicrob Chemother 2008 61 4 884 891 10.1093/jac/dkn032 18252692

[B11] Mills PR Davies RJ Devalia JL Airway epithelial cells, cytokines, and pollutants Am J Respir Crit Care Med 1999 160 5 Pt 2 S38 43 10556168 10.1164/ajrccm.160.supplement_1.11

[B12] Amsden GW Anti-inflammatory effects of macrolides--an underappreciated benefit in the treatment of community-acquired respiratory tract infections and chronic inflammatory pulmonary conditions? J Antimicrob Chemother 2005 55 1 10 21 10.1093/jac/dkh519 15590715

[B13] Tateda K Comte R Pechere JC Kohler T Yamaguchi K Van Delden C Azithromycin inhibits quorum sensing in Pseudomonas aeruginosa Antimicrob Agents Chemother 2001 45 6 1930 1933 10.1128/AAC.45.6.1930-1933.2001 11353657 PMC90577

[B14] Tateda K Standiford TJ Pechere JC Yamaguchi K Regulatory effects of macrolides on bacterial virulence: potential role as quorum-sensing inhibitors Curr Pharm Des 2004 10 25 3055 3065 10.2174/1381612043383377 15544497

[B15] Nalca Y Jansch L Bredenbruch F Geffers R Buer J Haussler S Quorum-sensing antagonistic activities of azithromycin in Pseudomonas aeruginosa PAO1: a global approach Antimicrob Agents Chemother 2006 50 5 1680 1688 10.1128/AAC.50.5.1680-1688.2006 16641435 PMC1472232

[B16] Kohler T Dumas JL Van Delden C Ribosome protection prevents azithromycin-mediated quorum-sensing modulation and stationary-phase killing of Pseudomonas aeruginosa Antimicrob Agents Chemother 2007 51 12 4243 4248 10.1128/AAC.00613-07 17876004 PMC2167979

[B17] Skindersoe ME Alhede M Phipps R Yang L Jensen PO Rasmussen TB Bjarnsholt T Tolker-Nielsen T Hoiby N Givskov M Effects of antibiotics on quorum sensing in Pseudomonas aeruginosa Antimicrob Agents Chemother 2008 52 10 3648 3663 10.1128/AAC.01230-07 18644954 PMC2565867

[B18] Saiman L Marshall BC Mayer-Hamblett N Burns JL Quittner AL Cibene DA Coquillette S Fieberg AY Accurso FJ Campbell PW 3rd Azithromycin in patients with cystic fibrosis chronically infected with Pseudomonas aeruginosa: a randomized controlled trial JAMA 2003 290 13 1749 1756 10.1001/jama.290.13.1749 14519709

[B19] Yates B Murphy DM Forrest IA Ward C Rutherford RM Fisher AJ Lordan JL Dark JH Corris PA Azithromycin reverses airflow obstruction in established bronchiolitis obliterans syndrome Am J Respir Crit Care Med 2005 172 6 772 775 10.1164/rccm.200411-1537OC 15976371

[B20] Labro MT Abdelghaffar H Immunomodulation by macrolide antibiotics J Chemother 2001 13 1 3 8 11233797 10.1179/joc.2001.13.1.3

[B21] Labro MT Interference of antibacterial agents with phagocyte functions: immunomodulation or "immuno-fairy tales"? Clin Microbiol Rev 2000 13 4 615 650 10.1128/CMR.13.4.615-650.2000 11023961 PMC88953

[B22] Bals R Beisswenger C Blouquit S Chinet T Isolation and air-liquid interface culture of human large airway and bronchiolar epithelial cells J Cyst Fibros 2004 3 Suppl 2 49 51 10.1016/j.jcf.2004.05.010 15463925

[B23] Shaykhiev R Beisswenger C Kandler K Senske J Puchner A Damm T Behr J Bals R Human endogenous antibiotic LL-37 stimulates airway epithelial cell proliferation and wound closure Am J Physiol Lung Cell Mol Physiol 2005 289 5 L842 848 10.1152/ajplung.00286.2004 15964896

[B24] Elssner A Jaumann F Dobmann S Behr J Schwaiblmair M Reichenspurner H Furst H Briegel J Vogelmeier C Elevated levels of interleukin-8 and transforming growth factor-beta in bronchoalveolar lavage fluid from patients with bronchiolitis obliterans syndrome: proinflammatory role of bronchial epithelial cells. Munich Lung Transplant Group Transplantation 2000 70 2 362 367 10.1097/00007890-200007270-00022 10933164

[B25] Shalit I Kletter Y Halperin D Waldman D Vasserman E Nagler A Fabian I Immunomodulatory effects of moxifloxacin in comparison to ciprofloxacin and G-CSF in a murine model of cyclophosphamide-induced leukopenia Eur J Haematol 2001 66 5 287 296 10.1034/j.1600-0609.2001.066005287.x 11422407

[B26] Barnes PJ Shapiro SD Pauwels RA Chronic obstructive pulmonary disease: molecular and cellular mechanisms Eur Respir J 2003 22 4 672 688 10.1183/09031936.03.00040703 14582923

[B27] Hackett TL Holloway R Holgate ST Warner JA Dynamics of pro-inflammatory and anti-inflammatory cytokine release during acute inflammation in chronic obstructive pulmonary disease: an ex vivo study Respir Res 2008 9 47 10.1186/1465-9921-9-47 18510721 PMC2435536

[B28] Vlahos R Bozinovski S Hamilton JA Anderson GP Therapeutic potential of treating chronic obstructive pulmonary disease (COPD) by neutralising granulocyte macrophage-colony stimulating factor (GM-CSF) Pharmacol Ther 2006 112 1 106 115 10.1016/j.pharmthera.2006.03.007 16716406

[B29] Baggiolini M Dewald B Moser B Human chemokines: an update Annu Rev Immunol 1997 15 675 705 10.1146/annurev.immunol.15.1.675 9143704

[B30] Nakamura H Yoshimura K Jaffe HA Crystal RG Interleukin-8 gene expression in human bronchial epithelial cells J Biol Chem 1991 266 29 19611 19617 1918068

[B31] Baldwin DR Andrews JM Wise R Honeybourne D Bronchoalveolar distribution of cefuroxime axetil and in-vitro efficacy of observed concentrations against respiratory pathogens J Antimicrob Chemother 1992 30 3 377 385 10.1093/jac/30.3.377 1452503

[B32] James NC Donn KH Collins JJ Davis IM Lloyd TL Hart RW Powell JR Pharmacokinetics of cefuroxime axetil and cefaclor: relationship of concentrations in serum to MICs for common respiratory pathogens Antimicrob Agents Chemother 1991 35 9 1860 1863 1952858 10.1128/aac.35.9.1860 PMC245281

[B33] Connors JE DiPiro JT Hayter RG Hooker KD Stanfield JA Young TR Assessment of cefazolin and cefuroxime tissue penetration by using a continuous intravenous infusion Antimicrob Agents Chemother 1990 34 6 1128 1131 2393271 10.1128/aac.34.6.1128 PMC171770

[B34] Perea EJ Ayarra J Garcia Iglesias MC Garcia Luque I Loscertales J Penetration of cefuroxime and ceftazidime into human lungs Chemotherapy 1988 34 1 1 7 10.1159/000238539 3280266

[B35] Soubani AO Uberti JP Bronchiolitis obliterans following haematopoietic stem cell transplantation Eur Respir J 2007 29 5 1007 1019 10.1183/09031936.00052806 17470622

[B36] Chien JW Martin PJ Gooley TA Flowers ME Heckbert SR Nichols WG Clark JG Airflow obstruction after myeloablative allogeneic hematopoietic stem cell transplantation Am J Respir Crit Care Med 2003 168 2 208 214 10.1164/rccm.200212-1468OC 12649126

[B37] Shinkai M Foster GH Rubin BK Macrolide antibiotics modulate ERK phosphorylation and IL-8 and GM-CSF production by human bronchial epithelial cells Am J Physiol Lung Cell Mol Physiol 2006 290 1 L75 85 10.1152/ajplung.00093.2005 16085674

[B38] Weiss T Shalit I Blau H Werber S Halperin D Levitov A Fabian I Anti-inflammatory effects of moxifloxacin on activated human monocytic cells: inhibition of NF-kappaB and mitogen-activated protein kinase activation and of synthesis of proinflammatory cytokines Antimicrob Agents Chemother 2004 48 6 1974 1982 10.1128/AAC.48.6.1974-1982.2004 15155187 PMC415605

